# A short update on the structure of drug binding sites on neurotransmitter transporters

**DOI:** 10.1186/1756-0500-4-559

**Published:** 2011-12-22

**Authors:** Mari Gabrielsen, Ingebrigt Sylte, Svein G Dahl, Aina W Ravna

**Affiliations:** 1 Medical Pharmacology and Toxicology Research Group, Department of Medical Biology, Faculty of Health Sciences, University of Tromsø, 9037 Tromsø, Norway

## Abstract

**Background:**

The dopamine (DAT), noradrenalin (NET) and serotonin (SERT) transporters are molecular targets for different classes of psychotropic drugs. Cocaine and the SSRI (*S*)-citalopram block neurotransmitter reuptake competitively, but while cocaine is a non-selective reuptake inhibitor, (*S*)-citalopram is a selective SERT inhibitor.

**Findings:**

Here we present comparisons of the binding sites and the electrostatic potential surfaces (EPS) of DAT, NET and SERT homology models based on two different LeuT_Aa _templates; with a substrate (leucine) in an occluded conformation (PDB id 2a65), and with an inhibitor (tryptophan) in an open-to-out conformation (PDB id 3f3a). In the occluded homology models, two conserved aromatic amino acids (tyrosine and phenylalanine) formed a gate between the putative binding pockets, and this contact was interrupted in the open to out conformation. The EPS of DAT and NET were generally negative in the vestibular area, whereas the EPS of the vestibular area of SERT was more neutral.

**Conclusions:**

The findings presented here contribute as an update on the structure of the binding sites of DAT, NET and SERT. The updated models, which have larger ligand binding site areas than models based on other templates, may serve as improved tools for virtual ligand screening.

## Introduction

There are three main dopaminergic pathways in the brain, the mesolimbic/mesocortical pathway involved in emotion- and drug-induced reward systems, the nigrostriatal pathway involved in motor control, and the tuberohypophyseal neurons involved in regulation of secretions from pituitary gland. The reward system is linked to drug abuse, and it is activated when a person receives positive reinforcement for certain behaviors, which can be artificial rewards (addictive drugs) or natural rewards (sex or food). When cocaine binds to DAT, a "reward" associated with an elevated dopamine concentration in the synapse in the Nucleus Accumbens occurs [1]. Individuals with the hypodopaminergic trait involving an impairment of the reward cascade ("Brain Reward Cascade Model") termed "Reward Deficiency syndrome" (RDS), which may be due to several genes, and also due to environmental stimuli, have higher risk for substance abuse, impulsive behavior, eating bingeing, pathological gambling, ADHD and sex addiction etc. [2,3].

Cocaine has similar binding affinities for DAT and the noradrenalin (NET) and serotonin (SERT) transporters. In addition to stimulant action, NET and SERT are molecular targets for antidepressants (selective serotonin reuptake inhibitors (SSRIs), selective noradrenaline reuptake inhibitors (NERI), serotonin-noradrenalin reuptake inhibitors (SNRI), and tricyclic antidepressants (TCA)). Binding studies have demonstrated that SSRIs are from 300 to 3,500 times more selective for SERT over NET, and generally have low affinities for DAT [4].

DAT, NET and SERT belong to the large neurotransmitter: sodium symporter (NSS) family of transporters [5], and they regulate monoamine concentrations at neuronal synapses by carrying monoamines across neuronal membranes into presynaptic nerve cells, using an inwardly directed sodium gradient as an energy source. Cocaine elevates the concentration of all three neurotransmitters at synapses, while SSRIs and NERIs elevate the concentration of serotonin and noradrenaline, respectively. Both serotonergic and noradrenergic neurons are localized in the pons and medulla (raphe nuclei), and their axons project to brain regions such as the limbic system, the cerebral cortex and hypothalamus.

Structural information about DAT, SERT and NET and their drug interactions is important for understanding their molecular mechanisms of action, and provide useful tools for new drug discovery. Elucidating differences in binding site conformations of occluded and open-outward neurotransmitter transporter models, and investigating differences in the electrostatic potential surfaces of the three transporters, may give insight into binding modes of drugs in different conformations and to the different transporters. No X-ray crystal structures of mammalian human DAT, SERT or NET have been reported, but several *Aquifex aeolicus *LeuT_Aa _crystal structures have been published [6-8]. LeuT_Aa _is a bacterial homologue of DAT, SERT and NET that is regarded as suitable template for molecular models of these transporters. The sequence identity between LeuTAa and DAT, NET and SERT is ~20% [9].

## Methods

In present study, we have used the crystal structure of LeuT_Aa_, complexed with an inhibitor (tryptophan) in an open-to-out conformation [6] (PDB id 3f3a) as a template for molecular modeling of DAT, SERT and NET. We have compared the binding site conformations of the models with the binding site conformations of our previous DAT, SERT and NET models [10] that were based on the crystal structure of LeuT_Aa_, complexed with a substrate (leucine) in an occluded conformation [7] (PDB id 2a65). Molecular models of DAT, NET and SERT were constructed using the ICM version 3.6 [11]. The modeling procedure of DAT, NET and SERT based on LeuT_Aa _in the occluded conformation [7] has previously been described in [10]. A comprehensive amino acid sequence alignment [9] of all known prokaryotic and eukaryotic neurotransmitter: sodium symporter (NSS) proteins including DAT, NET and SERT, was used as input alignment. In the ICM homology modeling module, the model is constructed from a few core sections defined by the average of C_α _atom positions in the conserved regions. Loops are searched for within several thousand high quality three dimensional (3D) protein structures by matching them with regard to sequence similarity and steric interactions with the surroundings of the model. The best fitting loop is selected by calculating the maps around the loops and scoring them based on their relative energies.

The models were refined by globally optimizing side-chain positions and annealing of the backbones using the RefineModel macro of ICM. This macro included (1) a Monte Carlo simulation [12] of side chains, (2) five steps of iterative annealing of the backbone structure, and (3) a second Monte Carlo simulation of side chains. In step 1 of the RefineModel macro, a side-chain conformational analysis using the "MonteCarlo fast" option of the ICM global optimization procedure for sampling of the conformational space of a molecule, is performed [12]. Iterative random movements, followed by local energy minimizations, and by a complete energy calculation, were performed, and each iteration was accepted or rejected based on energy and temperature criteria. Step 2 performed an iterative annealing of the backbone with provided tethers, which are harmonic restraints pulling an atom in the model to a static point in space represented by a corresponding atom in the template. A second Monte Carlo side-chain sampling was performed in step 3.

The ICM PocketFinder algorithm, which detects cavities of sufficient size to bind ligands, [13] was used to detect putative drug binding pockets in the DAT, NET and SERT models. A tolerance level of 3 was used, thus cavities with a volume greater than 3 Å^3 ^were considered. The reason for choosing a tolerance level of 3, instead of the default tolerance level of 4.6, is that a larger area is detected as a binding pocket. The algorithm, which uses a transformation of the Lennard-Jones potential calculated from a three-dimensional protein structure, does not require any knowledge about a potential ligand molecule. Thus, the ICM PocketFinder algorithm is based solely on protein structure [13]. The detected putative drug binding pockets were compared with detected putative drug binding pockets in DAT, NET and SERT in occluded conformation [10].

## Results and discussion

**Figure 1:**
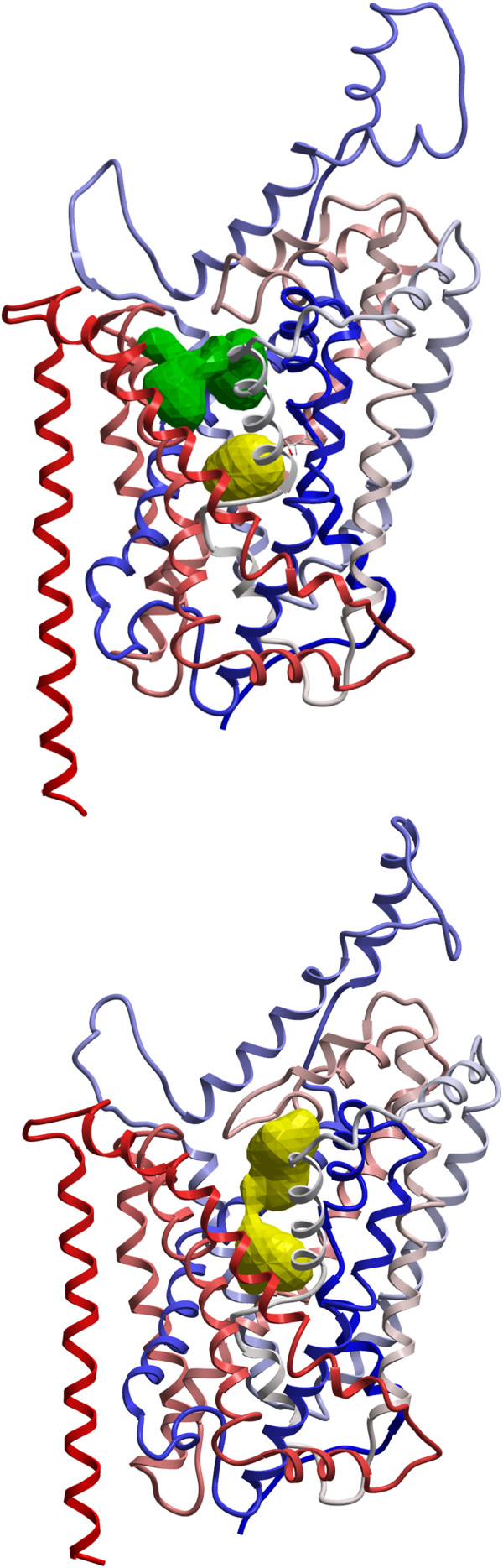
**Backbone Cα-traces of occluded DAT model **[10]**(A) and Open-to-out DAT model (B) viewed in the membrane plane cytoplasm downwards**. Binding sites as detected by ICM PocketFinder are displayed in yellow (S1) and green (S2) (A); and in yellow (B). Coloring of the C-alpha traces of the model is blue via white to red from N-terminal to C-terminal.

**Figure 2:**
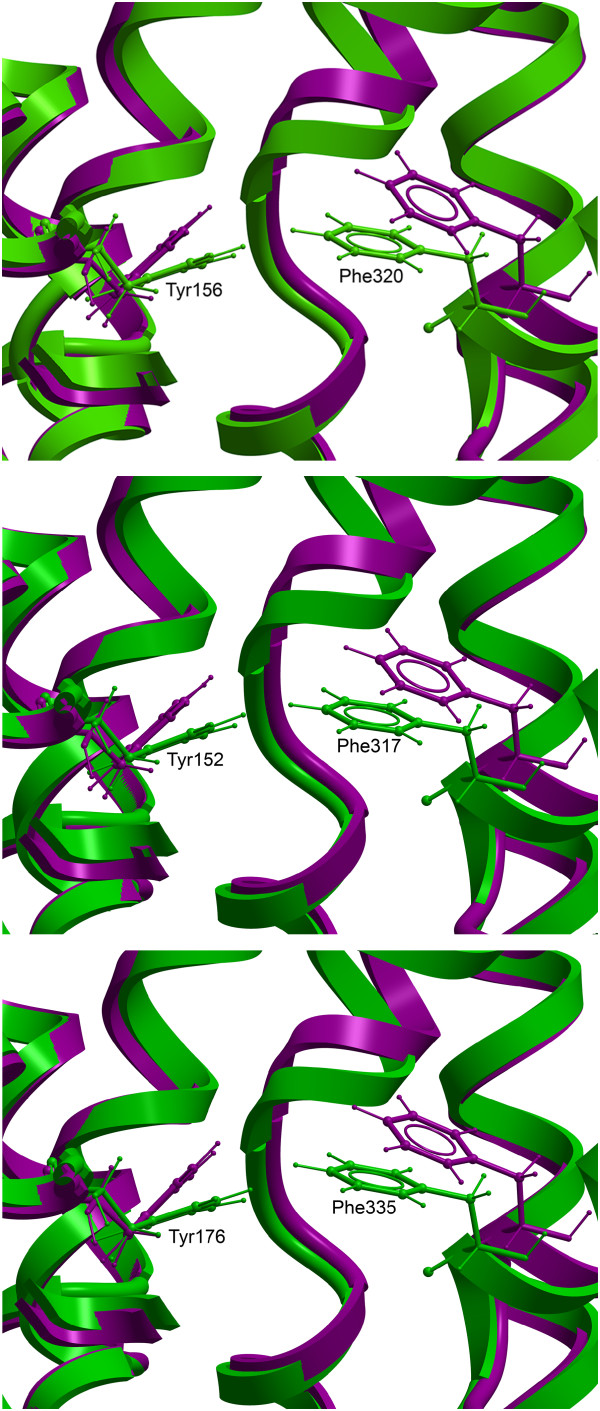
**The "Tyrosine-Phenylalanine Gate"**. Close-up view of differences in the "Tyrosine-Phenylalanine Gate" between the occluded (green) and open-to-out (purple) DAT (A), NET (B), and SERT (C) models.

The occluded and open-outward DAT models, with substrate binding pockets displayed, are shown in Figure 1. Two putative ligand binding pockets "S1" (substrate binding site) and "S2" (vestibular binding site) were detected in the occluded conformation, while in the open-to-out conformation the binding sites were "fused" and overlapping, yielding one large ligand binding pocket. Amino acids reported by ICM PocketFinder to contribute to the binding pockets of the open-to-out DAT, NET and SERT models are shown in Table 1. Two conserved aromatic amino acids (tyrosine and phenylalanine) formed a gate between the putative binding pockets in the occluded conformation, and the contact between these two amino acids was interrupted in the open to out conformation (Figure 2). Thus, when the transporter opens towards the extracellular side, these two amino acids part and the two binding sites merge into one large ligand binding area. Hindering the extracellular gate by separating the two amino acids with an aromatic moiety may be a mechanism for transporter inhibition. In a new and promising method for treating drug addicts, RDS is targeted by amino-acid precursor-enkephalinase therapy (NAAT), which in the long term activates dopamine, thus reducing vulnerability to relapse [2]. Interestingly, amino acids included in this neuronutritient include tyrosine and phenylalanine [14]. While these amino acids are precursors for dopamine, they could also, when administered in significant amounts, be interacting with the "Tyrosine-Phenylalanine Gate", thus temporarily blocking dopamine reuptake and increasing dopamine concentration at the synapse.

**Table 1:** Amino acids reported by ICM PocketFinder to contribute to the binding pockets of open-to-out DAT, NET and SERT

	DAT	NET	SERT
TMH1	F76, A77, D79, L80 A81, W84, R85, Y88	F72, A73, D75, L76, A77, W80, R81, Y84	Y95, A96, D98, G100, W103, R104, Y107, I108
TMH3	V152, F155, Y156, I159	145, V148, Y151, Y152, I155, W158	A169, I172, A173, Y175, Y176, I179, W182
TMH6	F320, S321, G323, F326, V328	F317, S318, G320, F323, V325	F335, L336, L337, G338, F341, V343
EL4	V382, A383, K384, D385, G386, P387, L389, I390	A380, E382, G383, A384, L386, V387, F388	A398, K399, D400, A401, P403, L405, L406, F407
TMH8	S422, A423, G426	S419, S420, G423	S438, T439, G442
TMH10	F472, D476, A480	G465, I466, L469, T470	E493, T497

The stereochemical qualities of the models were checked using the Structural Analysis and Verification Server (SAVS) http://nihserver.mbi.ucla.edu/SAVS/. Programs run on SAVS, which examine the stereochemical quality of a protein structure by analyzing its overall and residue-by-residue geometry, were Procheck [15], What_check [16], and Errat [17]. The overall quality factors of the DAT, NET and SERT models were 90.0, 93.7 and 87.6, respectively. The DAT model Ramachandran plot revealed that 93.8% of the residues were in the most favored regions, 5.9% were in additional allowed regions, 0.3% were in generously allowed regions, and 0.0% were in disallowed regions. The Ramachandran plot of NET reported 93.7% (most favored regions), 6.1% (additional allowed regions), 0.3% (generously allowed regions), and 0.0% (disallowed regions), and the Ramachandran plot of SERT reported 94.0% (most favored regions), 6.0% (additional allowed regions), 0.0% (generously allowed regions), and 0.0% (disallowed regions). All three models were satisfactory according to What_check.

**Figure 3:**
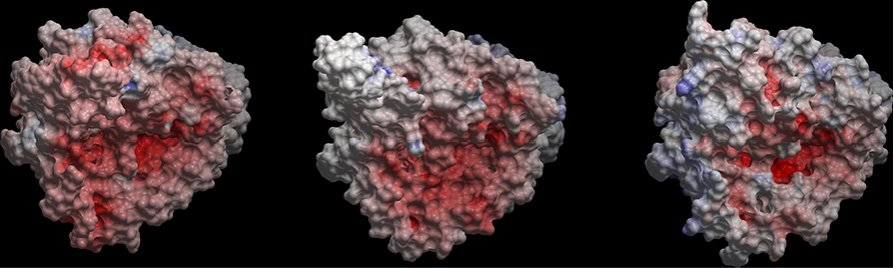
**EPS of open-to-out (purple) DAT (A), NET (B), and SERT (C) models viewed from the extracellular side**. Negative areas are colored red, positive areas are colored blue, and white areas are neutral. The entrance of S1 is seen in the lower right area, and S2 is localized above this area relative to the viewer.

The electrostatic potential surfaces (EPS) of the DAT, NET and SERT models were calculated with the ICM program, with a potential scale from -10 to +10 kcal mol^-1^. The EPS of the DAT, NET and SERT models viewed from the extracellular side are shown in Figure 3. While the EPS of DAT and NET were generally negative in the vestibular area, the EPS of the vestibular area of SERT was more neutral, with certain concentrated negative areas. The entrance into the S1 binding area is seen in the lower right area of each transporter. The S1 entrance of SERT was more clearly defined as a negative area than those of DAT end NET. These differences in electrostatics may partly explain the differences in drug recognition of DAT, NET and SERT.

The ligand binding site areas of our previous DAT, NET and SERT models [10] were tighter, possibly resulting in a greater chance of sterical hindrance for docked ligands. The updated models presented in this short report, which have larger ligand binding site areas than models based on other templates, may serve as improved tools for virtual ligand screening.

Co-ordinates of the DAT, NET and SERT models are available from the authors upon request.

## Competing interests

The authors declare that they have no competing interests.

## Authors' contributions

AWR carried out the molecular modeling studies (homology modeling and model refinement, and quality validation) and drafted the manuscript. MG, IS and SGD participated in the design of the study, and contributed with critical review of the manuscript. All authors read and approved the final manuscript.
